# *QuickStats:* Percentage* of Persons Who Had a Cold in the Past 2 Weeks,^†^ by Age Group and Calendar Quarter — National Health Interview Survey,^§^ United States, 2018

**DOI:** 10.15585/mmwr.mm6914a5

**Published:** 2020-04-10

**Figure:**
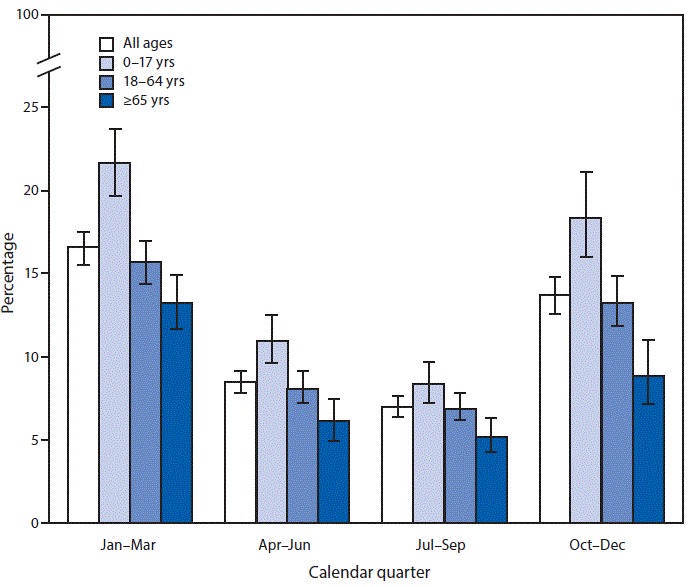
In 2018, the percentage of persons of all ages who had a cold during the past 2 weeks was 16.6% in January–March, 8.5% in April–June, 7.0% in July–September, and 13.7% in October–December. Across all calendar quarters, colds were more common in younger persons than in older persons. A higher percentage of persons in each age group had colds in the past 2 weeks in January–March and October–December than had colds in April–June or July–September 2018.

**Source:** National Health Interview Survey, 2018 data. https://www.cdc.gov/nchs/nhis.htm.

